# Paraneoplastic Focal Segmental Glomerulosclerosis in Association With Colon Cancer: A Rare Clinical Case Report

**DOI:** 10.1002/ccr3.71231

**Published:** 2025-10-10

**Authors:** Nida Fatima Daterdiwala, Hina Fatima Memon, Ambreen Bari, Sajjad Ali, Mahfuza Anan, Asif Husain Osmani

**Affiliations:** ^1^ Department of Medicine, Ziauddin Medical College Ziauddin University Karachi Pakistan; ^2^ Department of Histopathology Dr. Ziauddin Hospital Karachi Pakistan; ^3^ Department of Medicine Bangladesh Medical College Dhaka Bangladesh; ^4^ Department of Oncology Dr Ziauddin Hospital Karachi Pakistan

**Keywords:** cancer patients, colonic neoplasm, focal sclerosing glomerulonephritis, nephrotic syndrome, paraneoplastic syndrome

## Abstract

In patients with malignancy, the development of new‐onset nephrotic syndrome should prompt consideration of paraneoplastic focal segmental glomerulosclerosis and prompt early renal biopsy, guiding integrated oncologic and nephrologic management even when proteinuria persists. Timely diagnosis may influence both cancer treatment and renal outcomes, enhancing patient care despite advanced disease.

## 1 Introduction

Paraneoplastic syndromes are rare disorders resulting from the immune system's reaction to malignancies. Their manifestations vary widely, influenced by the type of cancer and the characteristics of the patient [1], occurring in approximately 7%–10% of cancer patients worldwide [2]. Membranous nephropathy (MN) is the most prevalent type of tumor‐associated nephropathy, accounting for 44%–49% of cases. The exact mechanism linking malignancy to glomerular disease is unclear, but it may involve autoantibodies targeting tumor antigens similar to glomerular components, causing damage through immune complex formation [1]. Focal segmental glomerulosclerosis (FSGS) is, therefore, an extremely rare presentation and has been reported in association with a limited number of cancers, some of which include renal cell carcinoma, invasive thymoma, and cancers of the lung [3]. Here, we report a unique case of a 79‐year‐old female with a known case of metastatic colon carcinoma (stage IV) who gradually developed associated FSGS. She underwent multiple cycles of chemotherapy to eradicate the malignancy. The nephrology department was taken on board to assist with the management of FSGS.

## 2 Case History

A 79‐year‐old female, with a history of chronic diabetes mellitus and hypertension (HTN), presented in the Out Patient Department (OPD) of Ziauddin Hospital, Karachi, Pakistan, on 18th October 2022. She had already been diagnosed with Stage 4 Carcinoma of the colon (involving the descending + sigmoid part) with peritoneal carcinomatosis two and a half months earlier. The patient now reported complaints of decreased appetite for 2 months, bilateral lower extremity swelling for 15 days, along with facial puffiness and shortness of breath on exertion for 3 days. She was electively admitted due to the above‐mentioned complaints.

A comprehensive assessment of the patient revealed normal vital parameters with findings suggestive of generalized body weakness and swelling extending up to the lower abdomen, along with pedal edema affecting both extremities. Bilateral crepitations were audible on chest auscultation, accompanied by decreased air entry on the left lower lobe of the lung. The abdomen was soft, non‐tender, and appeared distended. The rest of the examination was unremarkable. Further workup disclosed an albumin level of 1.21g/dL, indicating severe hypoalbuminemia. Other notable findings included the presence of blood and 150 mg/dL (+3) albumin on urinalysis. Urine protein and urine creatinine ratio were found to be 3.358. The complete blood count was normal, except for hemoglobin, which came out to be 10.5 g/dL.

## 3 Radiological Investigations and Differential Diagnosis

Potential causes of FSGS, including genetic mutations, viral infections, and drug‐induced nephropathy, were carefully ruled out through comprehensive clinical evaluation and laboratory testing. Extensive screening for secondary causes such as HIV, diabetes, and HTN also yielded no contributing factors, narrowing the diagnosis to a potential association with colorectal cancer (CRC).

**FIGURE 1:**
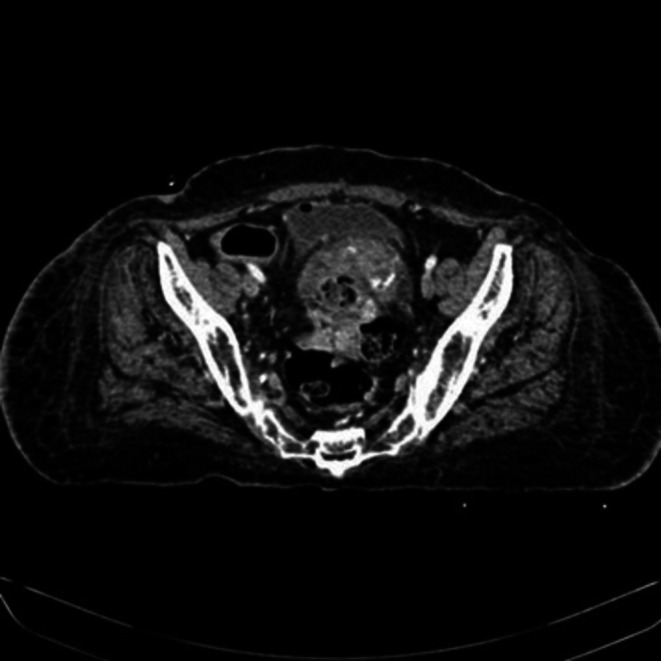
An axial view demonstrating a circumferential mass lesion within the sigmoid colon causing luminal narrowing, with a craniocaudal extent measuring approximately 7.5 cm and a single wall thickness of 1.9 cm.

Computed tomography (CT) scan of the chest, abdomen, and pelvis with and without contrast revealed irregular circumferential mural thickening engaging the sigmoid colon extending to involve the descending colon, ultimately causing partial luminal obliteration (Figure 1). A subserosal deposit along the proximal rectum indicated interval progression. Moreover, omental thickening and multiple nodular deposits were seen beneath the anterior abdominal wall. Nodular peritoneal thickening accompanied by peritoneal engorgement and a minor amount of ascites was also appreciated. Furthermore, tumor metastasis could be appreciated along the left ovary and its ligaments. A few lymph nodes were seen in the paraaortic and aortocaval locations as well as along the right common iliac vessels. A thrombus was detected in the right main pulmonary artery. There was no evidence of definite metastatic deposits in the bilateral lung fields.

**FIGURE 2:**
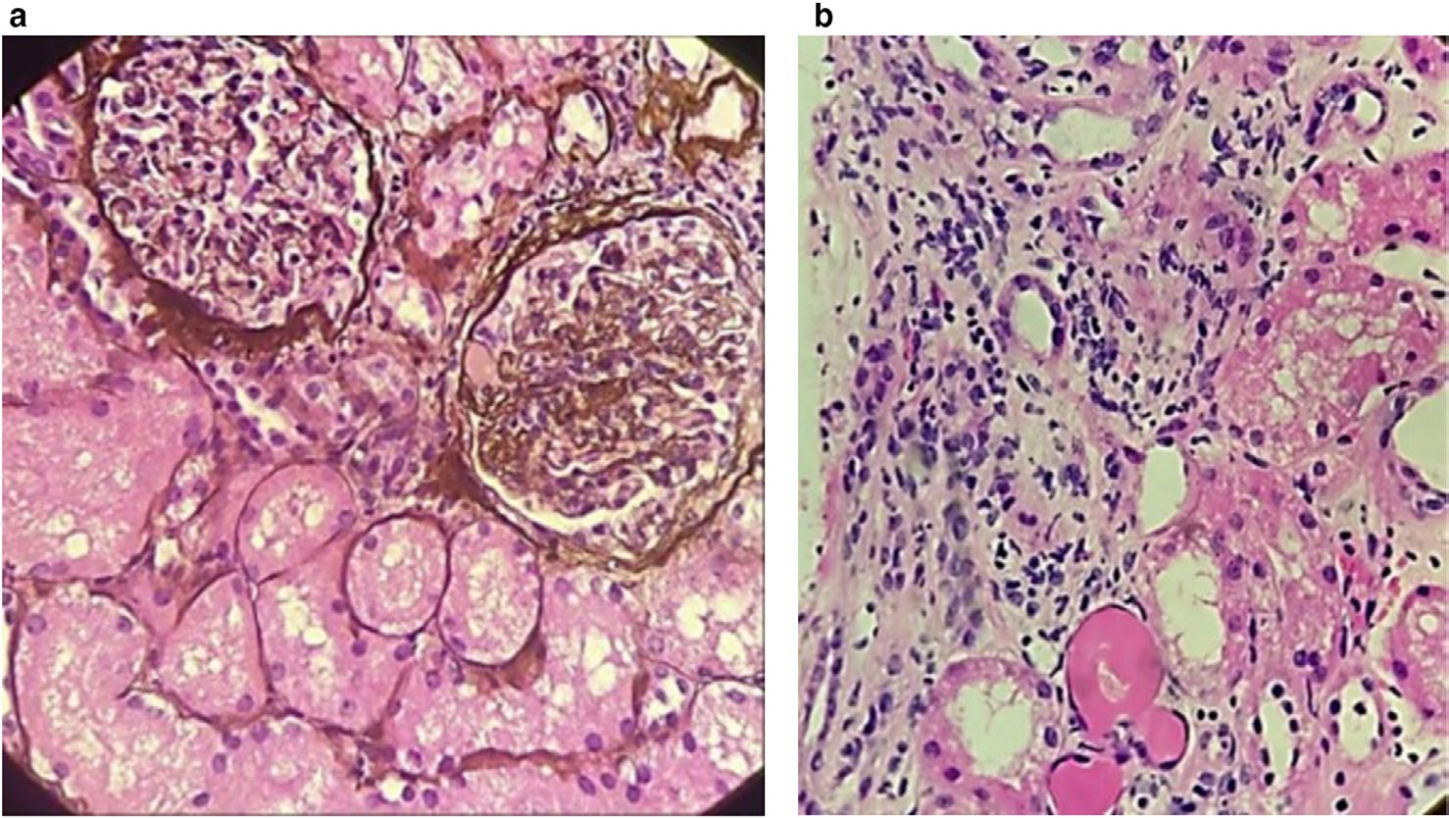
(a) Increased mesangial cellularity with mesangial cell interpositioning between GBM and endothelium, along with hyalinosis (GMS‐9; 40×). (b) Presence of lymphocytes, plasma cells, occasional eosinophils, and neutrophils in the interstitium (H&E; 10×).

CT and ultrasound scans of the kidneys, ureters, and bladder (CT KUB) displayed simple cortical cysts in both kidneys and mildly thickened urinary bladder walls. In addition, a renal biopsy was carried out, which unmasked features favoring focal segmental mesangiocapillary glomerulonephritis. The findings included the presence of fibrointimal thickening and hyalinosis along with inflammatory infiltrates (Figure 2a,b). Based on histopathological features, including endocapillary hypercellularity and inflammatory infiltrates, the biopsy findings are consistent with the cellular variant of FSGS, as described in the classification by D'Agati et al. [4].

## 4 Treatment and Outcome

The patient underwent six cycles of a chemotherapy regimen, which included Injection (Inj.) Cetuximab 415 mg, Inj. leucovorin 600 mg and Inj. 5‐fluorouracil 3000 mg. Additionally, IV albumin and Lasix were co‐administered multiple times to address bilateral pedal edema, and she was started on Inj. Hydrocortisone, which was tapered according to her blood glucose levels. Following treatment, a gradual increase in serum albumin was noted—from 1.21 to 2.3 g/dL—although this improvement is likely attributable to the exogenous albumin administration rather than intrinsic recovery of nephrotic syndrome. Unfortunately, serial urinalysis data revealed persistent proteinuria, with the urine protein‐to‐creatinine ratio remaining above 3.0. The patient succumbed to cancer and ultimately expired in September 2023.

## 5 Discussion

Paraneoplastic glomerular disease (PGD) evolves from cancer cell products, causing renal malfunction [5]. The exact mechanism by which malignancy leads to the development of glomerular disease remains unclear. However, one potential process involves the generation of autoantibodies that specifically target a tumor antigen sharing structural similarities with glomerular components, resulting in damage in that area through the formation of immune complexes [1]. Among paraneoplastic renal syndromes, MN is the most commonly reported, whereas FSGS is extremely rare, particularly in association with solid tumors.

FSGS is histologically defined by segmental sclerosis and collapse of the glomerular tuft in a focal pattern (i.e., affecting some but not all glomeruli). Clinically, it presents with features of nephrotic syndrome, which include proteinuria > 3.5 g/day, hypoalbuminemia, edema, hyperlipidemia, and progressive renal dysfunction. Although FSGS has occasionally been reported in conjunction with solid malignancies such as lung or renal cell carcinoma, to our knowledge, its association with CRC has not been previously documented in the medical literature [6].

Therefore, colorectal carcinoma screening should be done for early diagnosis and treatment. Most CRCs are sluggishly growing, arising from predecessor lesions such as adenomatous polyps or sessile serrated lesions. This slow expansion enables a window of time to screen for both premature malignancy and precursor lesions [7]. Early detection of colorectal carcinoma remains vital, as delayed diagnosis may increase the risk of rare paraneoplastic complications, including renal involvement such as FSGS [8].

Patients encountering glomerular proteinuria or Nephrotic Syndrome and malignancy are recommended to undergo renal biopsy depending on life expectancy and curative options [9]. A renal biopsy of our patient revealed findings consistent with Focal Segmental Mesangiocapillary Glomerulonephritis, and the case was treated accordingly.

The treatment for paraneoplastic glomerulonephritis focuses on addressing the underlying cancer, and it involves a team‐based approach to carefully monitor both the cancer and the kidney damage [10]. Studies have shown that the primary approach in addressing tumor‐associated nephropathy is curing the malignancy first. In instances where nephrotic syndrome persists despite cancer treatment or in cases with an inoperable tumor, immunosuppression may be considered as a substitute [10]. Hence, a thorough evaluation of the potential risks and benefits of immunosuppressive therapy is vital in the management of such cases.

## 6 Conclusion

In conclusion, FSGS‐associated CA colon is a rare manifestation among all types of nephrotic syndrome. This is the first case report being addressed here. Clinicians managing cancer patients with unexplained proteinuria should consider PGD as a potential cause. Given the nonspecific renal symptoms and the rarity of this association, a high index of suspicion and timely renal biopsy are essential for diagnosis. Despite supportive care and chemotherapy, renal involvement may persist and adversely affect outcomes. Early recognition may guide more comprehensive, multidisciplinary care and improve quality of life, even in advanced disease.

## Author Contributions

**Nida Fatima Daterdiwala:** formal analysis, investigation, project administration, supervision, validation, writing – original draft. **Hina Fatima Memon:** data curation, methodology, resources, software, writing – original draft. **Ambreen Bari:** formal analysis, investigation, methodology, resources, visualization, writing – original draft. **Sajjad Ali:** data curation, formal analysis, investigation, software, visualization, writing – review and editing. **Mahfuza Anan:** formal analysis, investigation, software, supervision, writing – review and editing. **Asif Husain Osmani:** conceptualization, project administration, supervision, validation, visualization, writing – review and editing.

## Ethics Statement

The authors have nothing to report.

## Consent

Written informed consent was obtained from the patient for their anonymized information to be published in this article.

## Conflicts of Interest

The authors declare no conflicts of interest.

## Data Availability

The data that support the findings of this study are available from the corresponding author upon reasonable request.
